# Association between extremely high-density lipoprotein cholesterol and adverse cardiovascular outcomes: a systematic review and meta-analysis

**DOI:** 10.3389/fcvm.2023.1201107

**Published:** 2023-06-27

**Authors:** Guanwei Zhang, Jiajuan Guo, Hongguang Jin, Xiaojing Wei, Xing Zhu, Weitao Jia, Yongsheng Huang

**Affiliations:** ^1^ College of Traditional Chinese Medicine, Changchun University of Chinese Medicine, Changchun, China; ^2^ Department of Cardiology, Affiliated Hospital of Changchun University of Chinese Medicine, Changchun, China; ^3^ Department of Geriatrics, Henan Provincial Veteran Cadres Rehabilitation Hospital, Zhengzhou, China; ^4^ Department of Internal Medicine, Longkou Traditional Chinese Medicine Hospital, Yantai, China

**Keywords:** high-density lipoprotein cholesterol, meta-analysis, cohort studies, adverse cardiovascular outcomes, the extremely high level

## Abstract

**Background:**

The association between high-density lipoprotein cholesterol (HDL-C) and adverse cardiovascular outcomes is understudied. Based on cohort studies, the current study aimed to investigate the association of extremely high HDL-C with all-cause, atherosclerotic cardiovascular disease (CVD) mortality, and stroke risk.

**Methods:**

A systematic literature search in Embase, PubMed, Cochrane Library, and Web of Science was performed to collect relevant cohort studies published before August 20, 2022. A random-effects model was used to pool relative risks (RRs) and 95% confidence intervals (CIs).

**Results:**

A total of 17 cohort studies involving 19,630,829 participants were included, encompassing 18,547,132 total deaths (1,328,036 CVD deaths). All-cause mortality, CVD mortality, and stroke risk in the extremely high HDL-C group were increased by 15% (RR = 1.15, 95% CI:1.05–1.25), 14% (RR = 1.14, 95% CI:0.96–1.35) and 14% (RR = 1.14, 95% CI:0.82–1.58), compared to the normal HDL-C group. In subgroup analyses, extremely high HDL-C was associated with a reduced risk of CVD mortality in women and a lower risk of stroke in men compared to normal HDL-C levels.

**Conclusions:**

The extremely high levels of HDL-C were associated with elevated risks of all-cause mortality, CVD mortality, and stroke. More well-designed studies are needed to confirm our findings.

**Systematic Review Registration:**

https://www.crd.york.ac.uk/PROSPERO/display_record.php?RecordID=370201, identifier: CRD42022370201.

## 1. Introduction

High-density lipoprotein cholesterol (HDL-C) has been identified as a risk factor for atherosclerotic cardiovascular disease (CVD), and a close association has been demonstrated between HDL-C and adverse cardiovascular outcomes (including all-cause mortality, cardiac vascular disease death, stroke, heart failure or myocardial infarction). Traditionally, HDL-C is believed to have an inverse correlation with CVD or mortality, and it is often called as “good cholesterol” (1–3). Unfortunately, its protective effect is increasingly questioned as the latest evidence accumulates gradually. A recent study found a non-linear dose-response relationship between HDL-C levels and cause-specific mortality, which is inconsistent with previous findings (4). An extremely high level of HDL-C paradoxically increases cardiovascular morbidity or mortality, rather than protecting the heart (4–7). In clinical trials, elevated HDL-C levels after cholesteryl ester transfer protein (CETP) inhibitor administration cannot reduce the incidence of cardiovascular events (8), and average high levels of HDL-C are intimately associated with a higher risk of overall stroke, ischemic stroke and hemorrhagic stroke (9). Furthermore, a heredity study indicated that high HDL-C levels did not reduce the risk of myocardial infarction (MI) (10). However, some published articles reported no significant association between HDL-C and all-cause mortality, cardiovascular death, stroke, and MI (11–13). Therefore, the clinical significance of HDL-C for assessing cardiovascular risk remains inconclusive and debatable.

However, HDL-C is still an essential variable in cardiovascular risk prediction models. Thus, the current study aimed to investigate the association between extremely high HDL-C and all-cause mortality, CVD mortality and stroke risk using a systematic review and meta-analysis based on cohort studies.

## 2. Materials and methods

### 2.1. Literature search

This systematic review was conducted in accordance with the Preferred Reporting Items for Systematic Reviews and Meta-Analyses. We performed a systematic literature search in Embase, PubMed, Cochrane Library, and Web of Science to collect cohort studies investigating the association between extremely high HDL-C levels and adverse cardiovascular outcomes published before August 20, 2022. The search terms included “HDL/High Density Lipoprotein Cholesterol” AND “Mortality/Death” OR “Stroke” OR “MACE” OR “Myocardial Infarction” OR “Heart Attacks” OR” Strokes” OR” Heart Failure” AND “Cohort Studies/prospective cohort/retrospective cohort”.

### 2.2. Inclusion and exclusion criteria

Cohort studies were included if they (a) assessed the association between extremely high HDL-C levels and risks of all-cause and cardiovascular death, stroke, MI, and heart failure; (b) reported relative risks (RR), hazard ratios (HR), or odds ratios (OR) of 95% confidence intervals (CI); (c) included estimates of at least three categories of HDL-C measurements in the analysis; (d) considered the normal levels of HDL-C as the reference range.

Meanwhile, the following studies were excluded: case-control studies, animal experiments, irrelevant outcomes, studies with no extremely high levels of HDL-C, duplicate publications, reviews, meta-analyses, non-English language, abstracts, letters, case reports, and articles for which the full texts were not available. Besides, if several studies were published based on an identical cohort, including HDL-C levels, we included the one that reported the most abundant data and had the largest sample sizes.

### 2.3. Data extraction and quality assessment

Two authors (Guanwei Zhang and Jiajuan Guo) extracted the following data: first authors, year of publication, geographic location, length of follow-up, sample size, participant gender, participant age, outcome type, stated HDL-C exposure levels, number of cases with HDL-C exposure of each category, total number or person/year of HDL-C exposure of each category, RR/HR/OR with 95% CI, and potential confounders adjusted in multivariate analysis. Any disagreements were resolved through discussion. The quality of included studies was assessed using the Newcastle-Ottawa Scale, which involved the selection of study groups, the comparability of the groups, and the ascertainment of outcomes of interest (14). Each study was scored up to nine 9 stars, and those scored >7 stars were considered as having high quality.

### 2.4. Statistical analysis

We assumed that the HR or OR for the risk of adverse cardiovascular outcomes was roughly the same as the RR (15) in the cohort study. Multivariate-adjusted RRs with 95% CI were extracted and used for data analysis. When heterogeneity (*I*^2^) < 50%, the combined RR and 95% CI were estimated using a fixed-effects model; otherwise, a random-effects model was applied. Subgroup analysis by gender was performed, and the data reported separately were pooled using a fixed-effects model.

Cochran Q and *I*^2^ statistics were applied to assess heterogeneity (16), and *P* < 0.05 was considered statistically significant. Subgroup analyses by gender and experimental design were conducted. Sensitivity analysis was performed by excluding one study at a time to assess whether the results were strongly influenced by a single study. Additionally, potential publication bias was assessed using Begg's and Egger's tests.

All analyses were performed using Stata 15.1 (Stata Corp, College Station, TX, USA), and a two-sided *P* < 0.05 was considered statistically significant.

## 3. Results

### 3.1. Literature retrieval and study characteristics

A total of 16 reports were included in this meta-analysis (7, 9, 13, 17–29), involving 17 cohort studies. The main characteristics of the included studies are presented in Table 1. Thirteen articles (7, 13, 17–19, 21–25, 28, 29) provided estimates of all-cause mortality; eleven articles (7, 13, 17, 18, 20–24, 28, 29) reported CVD mortality estimates; four (9, 21, 26, 27) reported estimates of stroke incidence, and one reported (21) estimates of MI incidence. Overall, this meta-analysis included 19 630 829 participants and 18 547 132 deaths (1 328 036 from CVD), with a median follow-up time over 7 years.

**Table 1:** Characteristics of studies included in the meta-analysis.

Author (year)	Country	Age (years)	Follow-up (years)	Sample size	Primary outcomes	Effect estimate	design	Subgroup	Comparison categories (mmol/L) and corresponding effect estimate (95% confidence interval)	Medicine using (lipid-lowering drugs)
Liu, C. (2022)	UK	40–72	8.9	14,478	All-cause mortality	HR	PC		1.96 (1.42–2.72)	NA
					CVD mortality				1.68 (1.07–2.63)	
Liu, C. (2022)	United States	≥18	6.7	5,467	All-cause mortality	HR	PC		1.63 (1.09–2.43)	YES
					CVD mortality				1.57 (0.95–2.61)	
Liu, C. (2022)	UK	37–73	19	415,416 187,465 227,951	All-cause mortality	HR	PC	All Men Women	1.12 (1.03–1.20) 1.80 (1.59–2.03) 0.96 (0.87–1.05)	NA
				415,416 187,465 227,951	CVD mortality			All Men Women	1.25 (1.06–1.48) 1.95 (1.54–2.46) 1.04 (0.82–1.31)	
Li, H (2022)	China	18–98	10	96,258	stroke	HR	PC		1.85 (1.63–2.09)	YES
Yi, S. W. (2021)	Korean	19–99	8.6	69,163	All-cause mortality	HR	PC	All Men Women	1.28 (1.22–1.35) 1.30 (1.23–1.38) 1.21 (1.11–1.31)	NA
Yang, Zong-Ming (2021)	China	≥40	8.71	176,243 167,444	All-cause mortality	HR	RC		0.98 (0.78–1.22) 1.23 (0.96–1.58)	YES
Yang, Yeoree (2021)	Korean	≥20	6	176,243 167,444	All-cause mortality		RC	Men Women	0.98 (0.78–1.22) 1.23 (0.96–1.58)	YES
					CVD mortality			Men Women	0.85 (0.44–1.66) 0.63 (0.30–1.35)	
					stroke			Men Women	0.93 (0.66–1.32) 1.11 (0.84–1.47)	
					MI			Men Women	0.75 (0.47–1.21) 0.98 (0.65–1.48)	
Huang, Yu-qing (2020)	United States	≥18	15	42,145 20,415 21,630	CVD mortality	HR	RC	All Men Women	0.99 (0.78,1.26) 1.15 (0.79–0.66) 0.90 (0.65–1.25)	YES
					All-cause mortality			All Men Women	1.14 (1.02,1.22) 1.43 (1.21–1.70) 1.02 (0.88–1.17)	
Chen, Chao-lei (2020)	United States	≥18	15	11,497	All-cause mortality	HR	RC	All Men Women	1.20 (1.06, 1.37) 1.47 (1.20, 1.81) 1.09 (0.92, 1.28)	YES
					CVD mortality			All Men Women	1.09 (0.83, 1.43) 1.11 (0.73, 1.70) 1.08 (0.75, 1.55)	
Oh, I. H. (2019)	Korean	>40	6	172,347 193,110	All-cause mortality	HR	RC	Men Women	1.17 (0.99–1.39) 1.12 (0.91–1.38)	NA
					CVD mortality			Men Women	0.69 (0.38–1.27) 0.71 (0.40–1.27)	
Li, X. (2019)	China	≥65, <98	8.76	100,070 79,913 20,157	All-cause mortality	HR	PC	All Men Women	Ref:1.00; 1.05 (0.93–1.18) Ref:1.00; 1.03 (0.92–1.17) Ref:1.00; 1.21 (0.80–1.81)	YES
Gu, Xiaoying (2019)	China	≥20	22	267,500	Ischemic Stroke	HR	PC		0.97 (0.89–1.06)	NA
					Hemorrhagic Stroke				0.94 (0.80–1.09)	
Hirata, A. (2018)	Japan	40–90	12	43,407 21,108 22,299	All-cause mortality	HR	RC	All Men Women	1.15 (0.90–1.46) 1.24 (0.92–1.66) 1.01 (0.67–1.54)	NA
					CVD mortality			All Men Women	1.43 (0.92–2.23) 1.65 (0.96–2.84) 1.13 (0.53–2.42)	
Saito, I. (2017)	Japan	40–69	15	10,571 20,165	Stroke	HR	PC	Men	Ref:1.00; 1.12 (0.90–1.41)	YES
								Women	Ref:1.00; 1.01 (0.80–1.27)	
Hirata, Aya (2016)	Japan	≥30	20	7,019 2,946 4,073	All-cause mortality	HR	PC	All Men Women	1.01 (0.80,1.27) 1.02 (0.73,1.43) 0.96 (0.69,1.32)	NA
				7,019 2,946 4,073	CVD mortality			All Men Women	1.14 (0.74,1.74) 1.08 (0.55,2.09) 1.20 (0.68,2.11)	
Ding, D. (2014)	China	40–85	5	1,916	All-cause mortality	HR	RC		3.06 (1.08–8.70)	YES
					CVD mortality				4.83 (1.47–15.8)	
Okamura, T. (2006)	Japan	≥30	9.6	8,384 3,504 4,880	All-cause mortality	HR	RC	All Men Women	0.70 (0.53, 0.93) 0.73 (0.50, 1.06) 0.63 (0.41, 0.94)	NA
				8,384 3,504 4,880	CVD mortality			All Men Women	0.56 (0.31, 1.01) 0.65 (0.29, 1.45) 0.48 (0.20, 1.15)	

CVD, cardiovascular disease; PC, prospective cohort; RC, retrospective cohort; HR, hazard ratio; Ref, reference.

In the entire cohort, all-cause mortality and CVD mortality were 9.05% and 6.8%, respectively, and all studies mainly involved adult populations. A total of seventeen studies included both females and males; eleven were stratified by gender (7, 13, 17, 19, 21–25, 27, 28) and one was analyzed by stroke type. Of the seventeen studies, two were from UK (17, 18), three from the US (17, 22, 23), three from Korea (19, 21, 24), four from Japan (7, 13, 27, 28), and five from China (9, 20, 25, 26, 29). Of these studies, nine mentioned the adjustment for variables such as lipid-lowering drug usage, and eight did not report information on drug usage. Analysis of study quality yielded a mean score greater than 7 on the Newcastle-Ottawa scale (ranging from 7 to 9).

### 3.2. Extremely high HDL-C levels and risk of all-cause mortality

**Figure 1:**
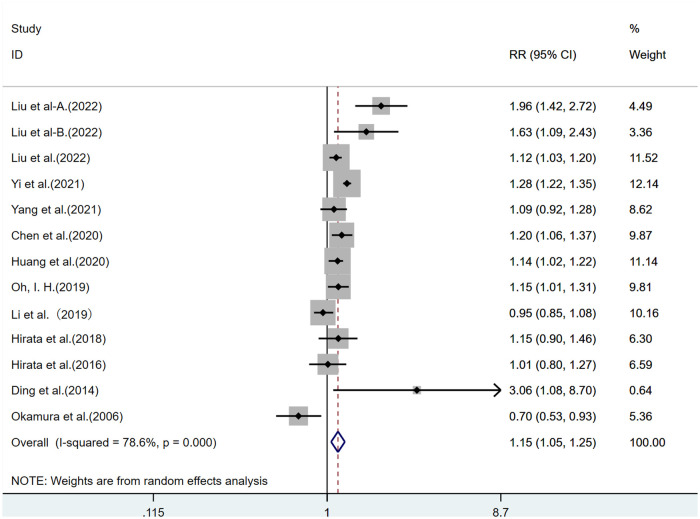
Risk of all-cause mortality for extremely high HDL-C levels. (CI: confidence interval; RR: relative risk. (**A and B**) in Liu et al-A and Liu et al-B in the figure refer to different cohort studies by Liu et al., involving different regions and populations, with independent cohort study results.).

A total of 13 publications were included in the binary analysis. Extremely high levels of HDL-C were associated with a 15% increased risk of all-cause mortality, compared with normal HDL-C (RR = 1.15, 95% CI: 1.05–1.25), with significant heterogeneity (*I*^2^ = 78.6%, Pheterogeneity < 0.001, Figure 1), as shown in Tables 1, 2.

**Table 2:** Subgroup analysis of pooled HRs and 95% CIs in all-cause mortality, CVD mortality, stroke.

Variable	No. of studies	No. of patients	Effects model	HR (95% CI)	*P*	Heterogeneity		*P* (begg)	*P* (egger)
						*I^2^*,% *P*			
All-cause mortality									
Total	10		Random	1.15 (1.05,1.25)	0.002	78.6	0.000	0.502	0.693
SEX									
Men	7	10,32,792		1.21 (1.05,1.39)	0.008	87.1	0.000		
Women	7	859,992		1.04 (0.95,1.15)	0.416	62.0	0.005		
Design									
RC	8	802,980		1.10 (1.00, 1.21)	0.059	59.3	0.031		
PC	5	16,41,6,116		1.23 (1.06, 1.42)	0.005	85.1	0.000		
CVD mortality									
Total	12	13,28,036	Random	1.14 (0.96,1.35)	0.127	60.7	0.003	0.304	0.826
SEX									
Men	8	5,89,737		1.14 (0.85, 1.54)	0.388	67.8	0.003		
Women	8	6,47,175		0.96 (0.83,1.12)	0.622	0.0	0.489		
Design									
RC	6	8,72,243		0.99 (0.81,1.22)	0.951	57.5	0.028		
PC	6	4,55,793		1.42 (1.11,1.82)	0.005	42.9	0.136		
Stroke									
Total	4	7,38,181	Random	1.14 (0.82,1.58)	0.432	96.7	0.000	0.308	0.666
SEX									
Men	2	3,52,486		0.89 (0.80,1.00)	0.057	0.0	0.815		
Women	2	1,87,609		1.04 (0.87,1.24)	0.691	0.0	0.535		
Design									
RC	1	3,43,687		1.04 (0.83, 1.30)	0.435	97.8	0.000		
PC	3	3,94,494		1.17 (0.79, 1.74)	0.727	96.7	0.000		

CVD, cardiovascular disease; PC, prospective cohort; RC, retrospective cohort; HR, hazard ratio; CI, confidence interval.

Furthermore, to explore the source of heterogeneity, we performed subgroup analyses. In the subgroup analysis by gender, the risk of all-cause mortality of extremely high HDL-C was 1.21 times higher than that of normal HDL-C (RR = 1.21, 95% CI: 1.05–1.39) in men, and extremely high levels of HDL-C had a 1.04-fold higher risk of all-cause mortality than normal HDL-C (RR = 1.04, 95% CI: 0.94–1.15) in women. Both had significant heterogeneity (*I*^2^ > 50%, Pheterogeneity < 0.001, Supplementary Figure S1). In the subgroup analysis by Asian and Western populations, the risk of all-cause mortality of extremely high HDL-C was 1.24 times higher than that of normal HDL-C (RR = 1.24, 95% CI:1.10–1.40) in the Western population, while the risk of all-cause mortality of extremely high HDL-C is 1.04 times that of normal HDL-C (RR = 1.07, 95% CI:0.93–1.23) in the Asian population. Both had significant heterogeneity (*I*^2^ > 50%, Pheterogeneity < 0.001, Supplementary Figure S2). In the subgroup analysis by trial design, there was no substantial difference in the risk of all-cause mortality (Supplementary Figure S3).

### 3.3. Extremely high HDL-C levels and risk of CVD mortality

**Figure 2:**
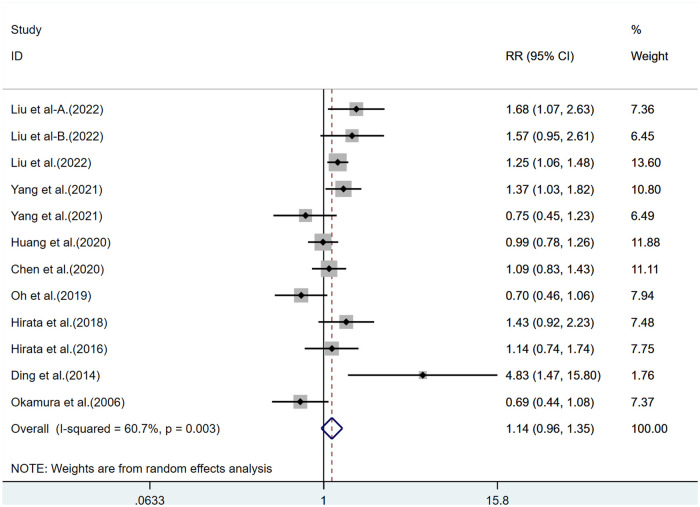
CVD mortality risk of extremely high HDL-C levels. (CVD: cardiovascular disease; CI: confidence interval; RR: relative risk.).

A total of 10 publications were included. The extremely high HDL-C group had a 14% increased risk of CVD mortality than the normal HDL-C group (RR = 1.14, 95% CI: 0.96–1.35), with significant heterogeneity (*I*^2^ = 60.7%, Pheterogeneity < 0.005, Table 2, Figure 2).

In the subgroup analysis by gender, extremely high levels of HDL-C were associated with a 14% increased risk of CVD mortality in men as compared with normal HDL-C levels (RR = 1.14, 95% CI: 0.85–1.54), with significant heterogeneity (*I*^2^ > 50%, Pheterogeneity < 0.005); Meanwhile, extremely high levels of HDL-C were associated with a 4% lower risk of CVD mortality in women as compared with normal HDL-C levels (RR = 0.96, 95% CI: 0.83–1.12), with low heterogeneity (*I*^2^ < 50%, Pheterogeneity > 0.001, Supplementary Figure S4). In the subgroup analysis by Asian and Western populations, extremely high levels of HDL-C were associated with an elevated risk of all-cause mortality in both Asian and Western populations. The heterogeneity was low in the Western population (*I*^2 ^< 50%, Pheterogeneity > 0.001) but significant in the Asian population (*I*^2^ > 50%, Pheterogeneity < 0.005, Supplementary Figure S5). In the subgroup analysis by trial design, the risk of all-cause mortality in the extremely high HDL-C group was significantly increased in prospective cohort studies but decreased in retrospective cohort studies (Supplementary Figure S6).

### 3.4. Extremely high HDL-C levels and stroke risk

**Figure 3:**
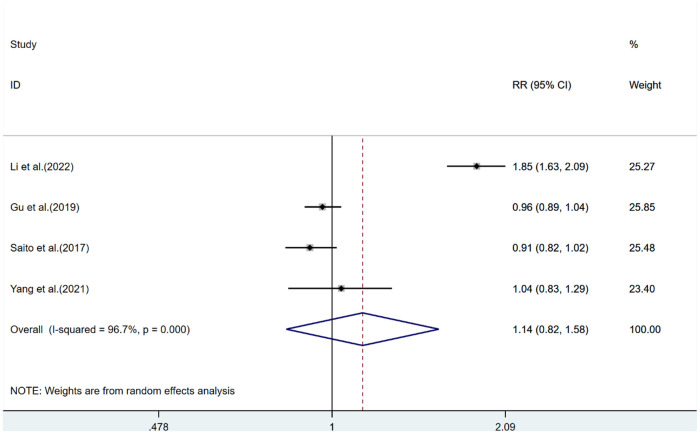
Stroke risk analysis for extremely high HDL-C levels. (CI: confidence interval; RR: relative risk.).

Four studies were included. Extremely high HDL-C was associated with a 14% increased risk of stroke (RR = 1.14, 95% CI: 0.82–1.58) as compared with normal HDL-C levels, with significant heterogeneity (*I*^2^ = 60.7%, Pheterogeneity < 0.001, Figure 3).

In the subgroup analysis by gender, extremely high levels of HDL-C were associated with a lower risk of stroke in men (RR = 0.89, 95% CI: 0.80–1.00) compared with normal HDL-C levels, with relatively low heterogeneity (*I*^2^ < 50%, Pheterogeneity > 0.001); women with extremely high levels of HDL-C had a stroke risk of 1.04 as compared to those with normal HDL-C (RR = 1.04, 95% CI: 0.87–1.24), with low heterogeneity (*I*^2^ < 50%, Pheterogeneity > 0.001, Supplementary Figure S7). In subgroup analysis by trial design, the risk of stroke increased in both groups (Supplementary Figure S8).

### 3.5. Sensitivity analysis and publication bias

**Figure 4:**
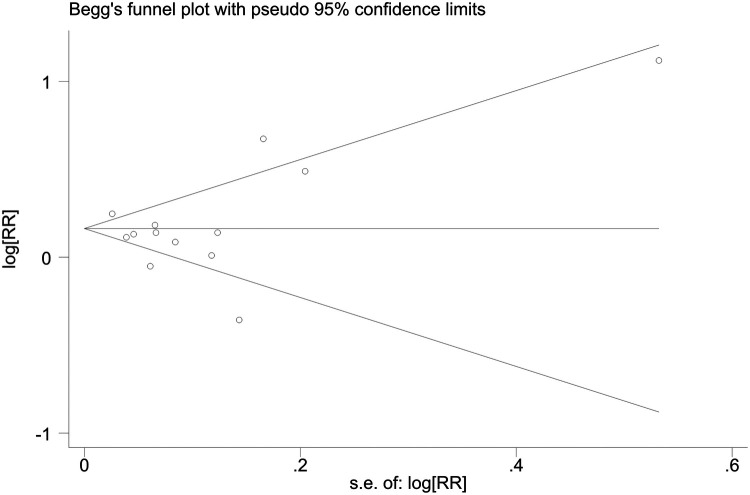
Begger's test for all-cause mortality at extremely high levels of HDL-C.

**Figure 5:**
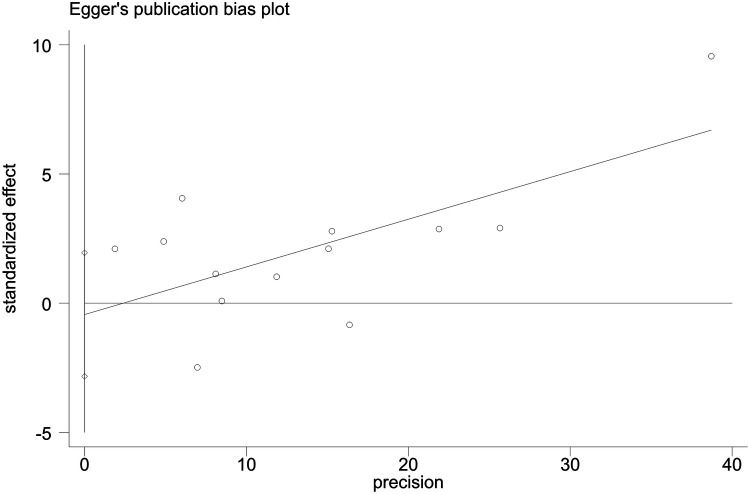
Egger's test for all-cause mortality at extremely high levels of HDL-C.

**Figure 6:**
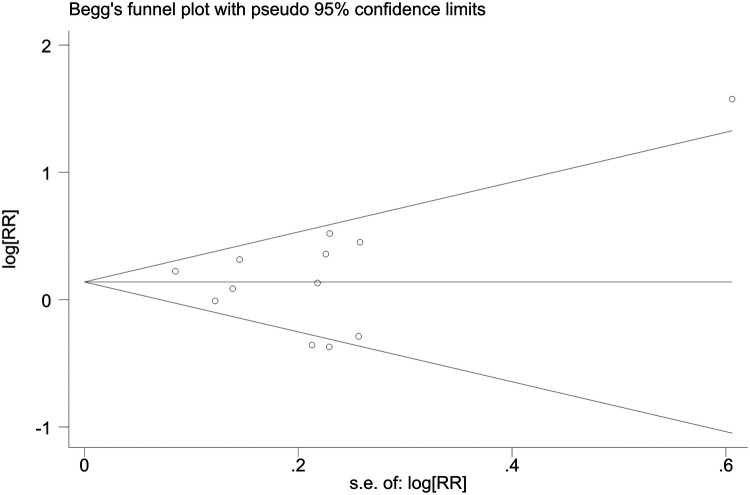
Begger's test for CVD death with extremely high levels of HDL-C.

**Figure 7:**
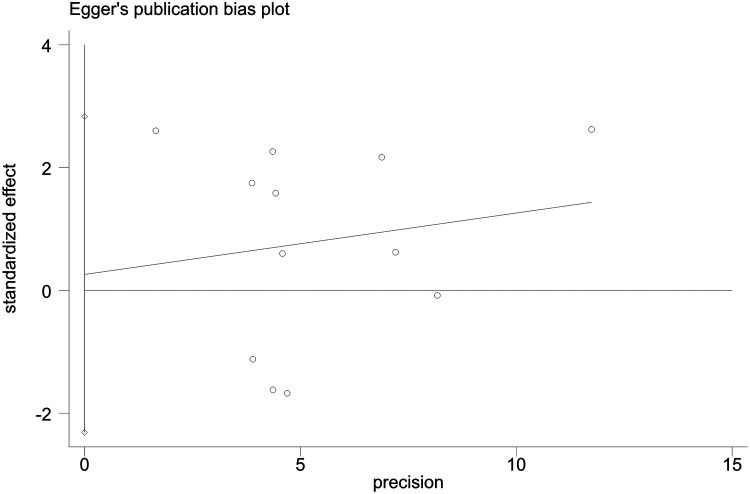
Egger's test for CVD death with extremely high levels of HDL-C.

**Figure 8:**
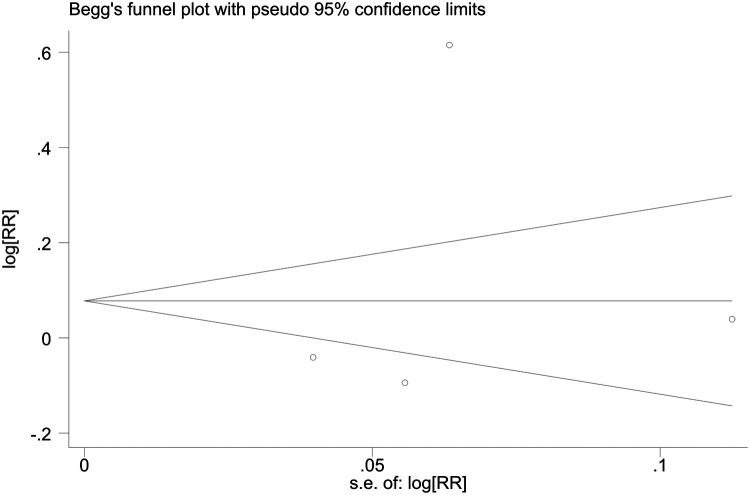
Begger's test for stroke with extremely high levels of HDL-C.

**Figure 9:**
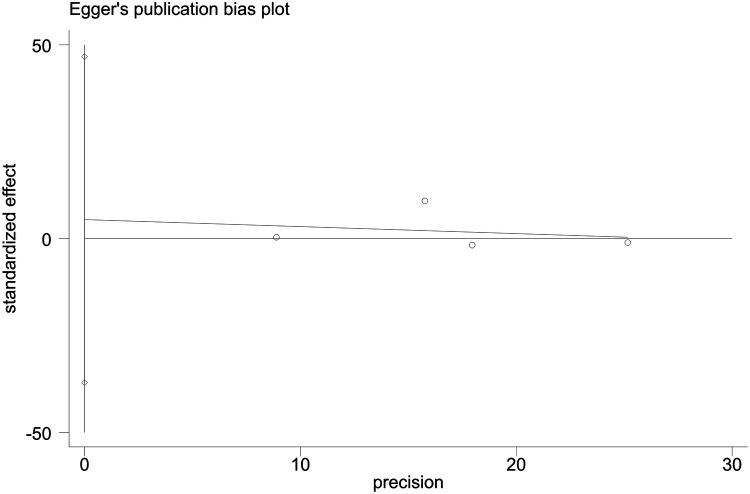
Egger's test for stroke with extremely high levels of HDL-C.

Sensitivity analyses indicated consistent results after the exclusion of each individual study (Supplementary Figures S9–S11). Neither Begger's test nor Egger's test revealed evidence of publication bias. For all-cause mortality of extremely high levels of HDL-C, Begger's test indicated *P* = 0.502, and Egger's test showed *P* = 0.693, as shown in Figures 4, 5. For CVD death of extremely high levels of HDL-C, Begger's test indicated *P* = 0.304 and Egger's test showed *P* = 0.826, as shown in Figures 6, 7. For the risk of stroke, Begger's test indicated *P* = 0.308 and Egger's test showed *P* = 0.666, as shown in Figures 8, 9.

## 4. Discussion

### 4.1. Summary of evidence

The current meta-analysis comprehensively assessed the association between extremely high HDL-C levels and adverse cardiovascular outcomes based on cohort studies. The results revealed that the risks of all-cause mortality, CVD mortality, and stroke increased by 15%, 14%, and 14% in the extremely high HDL-C group, respectively. Subgroup analysis revealed that extremely high levels of HDL-C showed differences in CVD mortality and stroke risks in men and women. Women with extremely high HDL-C had a lower risk of CVD mortality, compared with those with normal HDL-C. However, men with extremely high HDL-C had a significantly higher risk of CVD mortality. Meanwhile, the risk of stroke in women was proportional to extremely high levels of HDL-C and was higher than that in men, and the risk of stroke in men was inversely correlated with extremely high levels of HDL-C. Asian and Western populations with extremely high levels of HDL-C had an increased risk of all-cause mortality and CVD mortality, according to the subgroup analysis of Asian and Western populations.

### 4.2. Recent studies on the association of extremely high levels of HDL-C-mortality with adverse cardiovascular outcomes

Extremely high levels of HDL-C are found to be associated with an increased risk of all-cause and CVD mortality and stroke; however, their association has not been fully elucidated. Some studies revealed a positive association between HDL-C and CVD mortality (4, 5, 30), whereas some studies indicated no significant association between the two (12, 13, 28, 31–33). A pooled analysis of nine Japanese cohort studies including 43,407 participants aged 40–89 indicated that extremely high levels of HDL-C were highly associated with increased risks of all-cause mortality, CHD mortality, and ischemia stroke (7). Furthermore, gender differences have also been reported in previous studies, and another pooled dataset from six cohorts, including 24,440 participants aged 54–60 years, showed that the mortality of men was proportional to the level of HDL-C as compared to normal HDL-C, whereas the mortality of women increased after > 70 mg/dl, and the incidence of coronary heart disease events decreased with the increase of HDL-C level (30). Besides, a pooled analysis of 17 Canadian cohorts including 631,762 participants aged 40–105 years has indicated that a higher risk of cardiac/non-cardiac mortality was observed in individuals with extremely high HDL-C levels, and such risks remained elevated after adjustment for alcohol drinking (4).

### 4.3. Implications for research and practice compared with previous meta-analyses

Previous meta-analyses on HDL-C mostly applied the lowest or high levels of HDL-C as the reference range. Conversely, the current study utilized the normal level as the reference range to investigate the relationship between extremely high levels of HDL-C and adverse cardiovascular outcomes. Thus, it is possible to more clearly analyze the impact of extremely high levels of HDL-C on adverse cardiovascular outcomes, in the hope of offering some guidance for the prevention of CVD. Furthermore, the current meta-analysis involved several countries and multiple age groups, and it included the general population as the major research subject. Several subgroup analyses were performed by gender of the participants (male and female), trial design (prospective and retrospective cohort studies), and different populations (Asian and Western populations). The results of this meta-analysis provided updated summary evidence of the association between extremely high HDL-C and all-cause mortality, CVD mortality, and stroke. Subgroup analyses indicated differences in CVD mortality and stroke risks between men and women. Further verification revealed that extremely high HDL-C exerted no protective effect on cardiovascular and cerebrovascular as compared with normal levels of HDL-C, providing a reference for minimizing the risk of all-cause mortality and CVD mortality by adjusting HDL-C levels.

### 4.4. Underlying mechanisms

As HDL-C can reversely transport cholesterol, it can effectively reduce the accumulation of excess cholesterol. Thus, the formation of atherosclerosis is avoided (34, 35), which produces a protective effect on the cardiovascular system. However, HDL-C may have a biphasic effect. As the level of HDL-C increases, the corresponding contents or functions of HDL-C may also change. This is the reason why a higher concentration of HDL-C is not always better. The specific mechanisms underlying how elevated HDL-C levels increase the risks of all-cause mortality, CVD mortality, and stroke are still unclear. Some studies have pointed out that it may be related to genetic variation, but other studies have shown that associated genes CETP, LIPC, SCARB1, and ABCA1 (5, 36–38) can not only elevate HDL-C concentrations but also increase the risk of adverse outcomes. Another explanation is the complicated composition of HDL-C. As the concentration of HDL-C increases, the particle size, number, shape, and function or content of HDL-C may change. Such differences can produce different effects on the predictive ability of vascular events. Besides, high concentrations may not only damage and accelerate endothelial cell senescence (10), but may also lead to the retention of low-density lipoprotein particles in the arterial intima, which in turn reversely accelerates the development of atherosclerosis (39). Mendelian genetic randomization has failed to demonstrate a causal relationship between HDL and coronary heart disease, and several pharmacological trials that applied increased HDL-C levels have failed to reduce the risk of ASCVD (40–43). Several pathological conditions, such as genetic inflammatory diseases, genetic alterations, liver diseases, and endocrine diseases, may be contributing factors to extremely high HDL-C levels and cardiovascular risk [1, 38]. The pathophysiological role of extremely high HDL-C levels in the risk of adverse cardiovascular outcomes remains to be fully elucidated.

### 4.5. Limitations

This study also has several limitations. First, given the observational studies included in this meta-analysis, it is impossible to demonstrate a causal relationship between extremely high HDL-C and adverse cardiovascular outcomes. Second, there were limited data on the relationship between extremely high HDL-C levels and the risk of MI and heart failure in previous general population-based cohort studies, it was hard for the current work to perform corresponding analyses. Finally, residual confounding bias may be unavoidable due to unmeasured and under-measured variables.

## 5. Conclusions

This systematic review and meta-analysis indicated that extremely high HDL-C levels were inversely associated with all-cause mortality, CVD mortality, and the risk of stroke. Lifestyle interventions and prevention and control strategies are necessary to minimize the risks of HDL-C-related all-cause mortality, CVD mortality, and stroke. More studies are warranted to validate the inverse role of extremely high HDL-C levels in the risk of death from all-cause and CVD. Furthermore, the range of extremely high levels of HDL-C or the optimal threshold needs further exploration, which has favorable guiding significance for reducing cardiovascular risk using HDL-C intervention.

## Data Availability

The original contributions presented in the study are included in the article/**Supplementary Material**, further inquiries can be directed to the corresponding author.

## Supplementary material

SUPPLEMENTARY FIGURE S1

Subgroup analysis of all-cause mortality risk in men and women with very high HDL-C levels.

SUPPLEMENTARY FIGURE S2

Subgroup analysis of all-cause mortality risk in Asian and Western populations with very high HDL-C levels.

SUPPLEMENTARY FIGURE S3

Prospective and retrospective cohorts subgroup analysis of all-cause mortality risk in patients with very high HDL-C levels.

SUPPLEMENTARY FIGURE S4

Subgroup analysis of CVD mortality risk in men and women with very high HDL-C levels.

SUPPLEMENTARY FIGURE S5

Subgroup analysis of CVD mortality risk in Asian and Western populations with very high HDL-C levels.

SUPPLEMENTARY FIGURE S6

Prospective and retrospective cohorts subgroup analysis of CVD mortality risk in patients with very high HDL-C levels.

SUPPLEMENTARY FIGURE S7

Subgroup analysis of stroke risk in men and women with very high HDL-C levels.

SUPPLEMENTARY FIGURE S8

Prospective and retrospective cohorts subgroup analysis of stroke risk in patients with very high HDL-C levels.

SUPPLEMENTARY FIGURE S9

Sensitivity analysis of all-cause mortality.

SUPPLEMENTARY FIGURE S10

Sensitivity analysis of CVD mortality.

SUPPLEMENTARY FIGURE S11

Sensitivity analysis of Stroke.

SUPPLEMENTARY TABLE S1

Systematic literature review search terms and strategy.

SUPPLEMENTARY TABLE S2

Characteristics of studies included in the meta-analysis.

SUPPLEMENTARY TABLE S3

Newcastle-Ottawa Scale table.
