# Supplementary material for: Association between extremely high-density lipoprotein cholesterol and adverse cardiovascular outcomes: a systematic review and meta-analysis
Source: Front Cardiovasc Med. 2023 Jun 27;10:1201107. doi: 10.3389/fcvm.2023.1201107 (PMC10333521; doi:10.3389/fcvm.2023.1201107)
Supplement: Supplementary file 1 [file Datasheet1.docx]

Supplementary Material

Association between extremely high-density lipoprotein cholesterol and adverse cardiovascular outcomes: a systematic review and meta-analysis

Guanwei Zhang, Jiajuan Guo, Hongguang Jin, Xiaojing Wei, Xing Zhu, Weitao Jia, Yongsheng Huang*

*** Correspondence:** Yongsheng Huang: lingxi1622@163.com

# Supplementary Figures and Tables

## Supplementary Figures


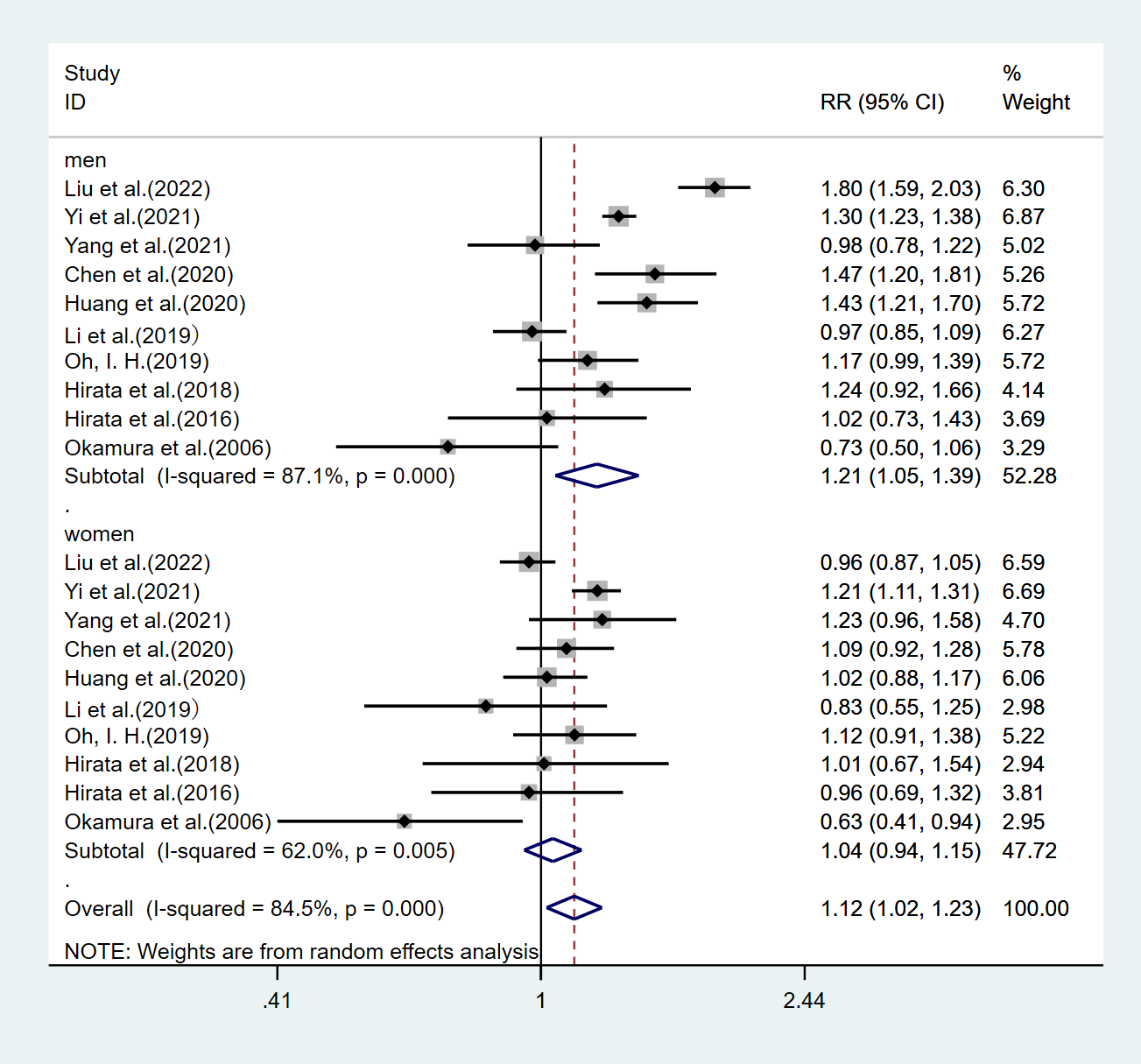


**Supplementary Figure S1.** Subgroup analysis of all-cause mortality risk in men and women with very high HDL-C levels.


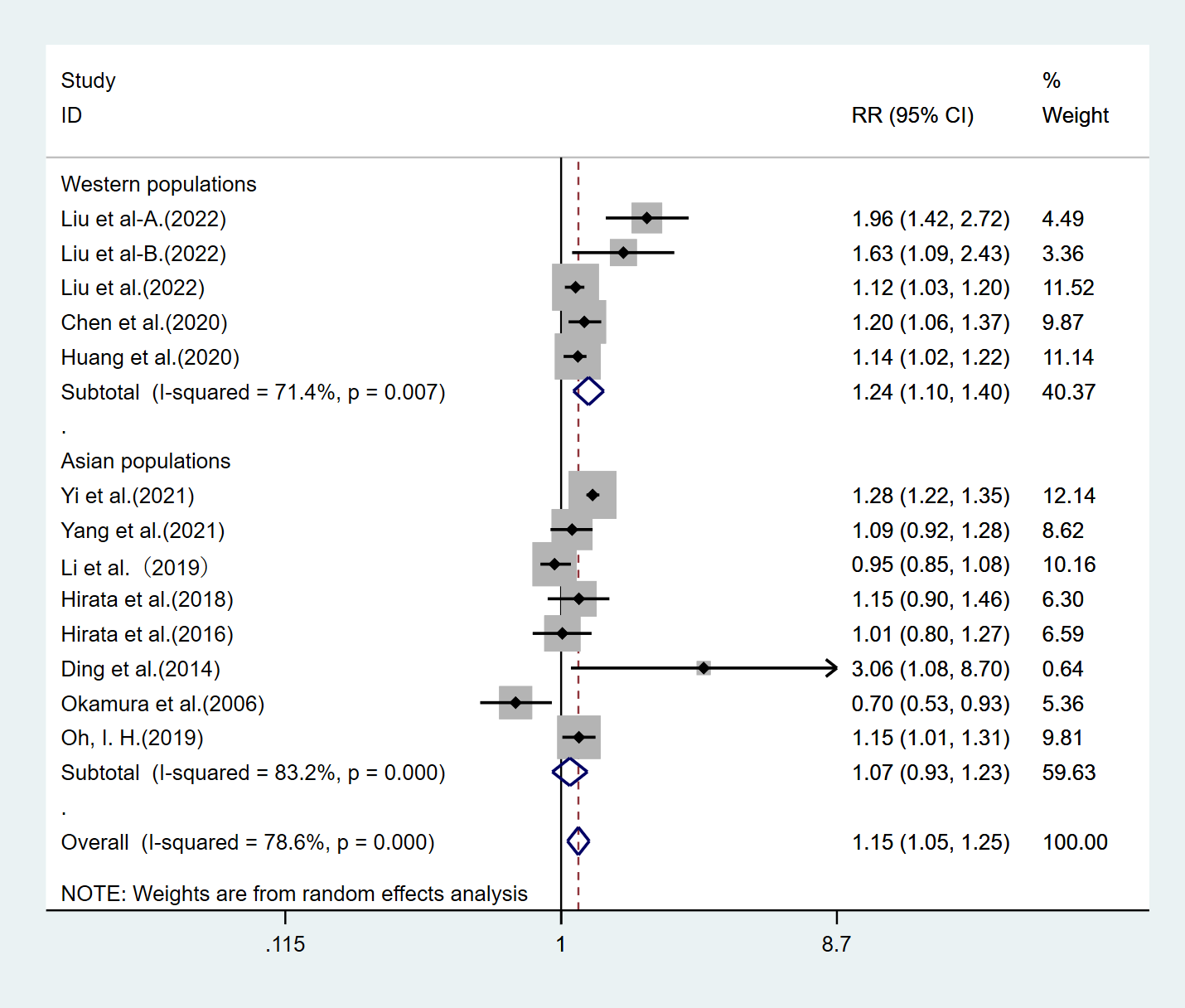


**Supplementary Figure S2.** Subgroup analysis of all-cause mortality risk in Asian and Western populations with very high HDL-C levels.


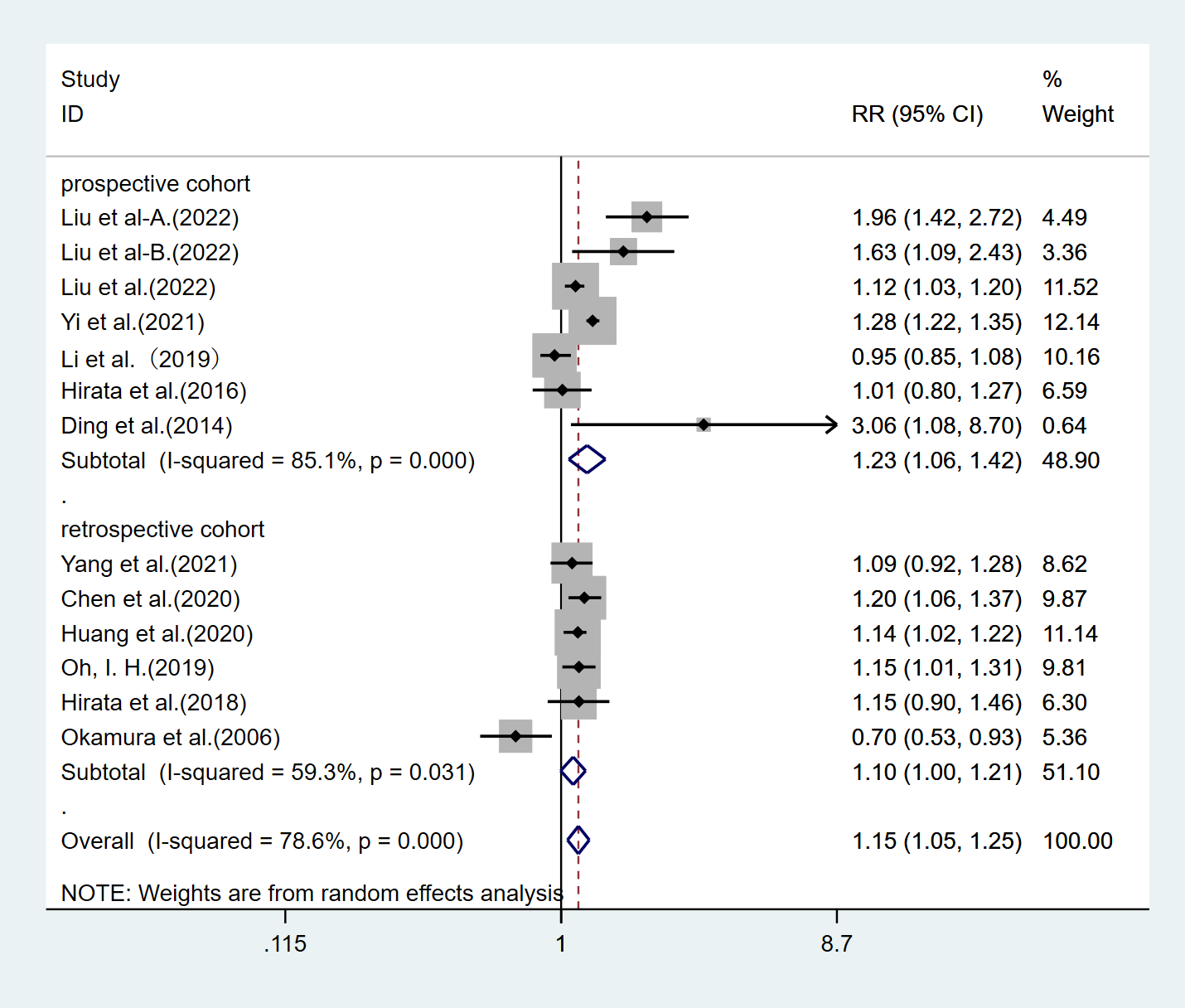


**Supplementary Figure S3.** Prospective and retrospective cohorts subgroup analysis of all-cause mortality risk in patients with very high HDL-C levels.


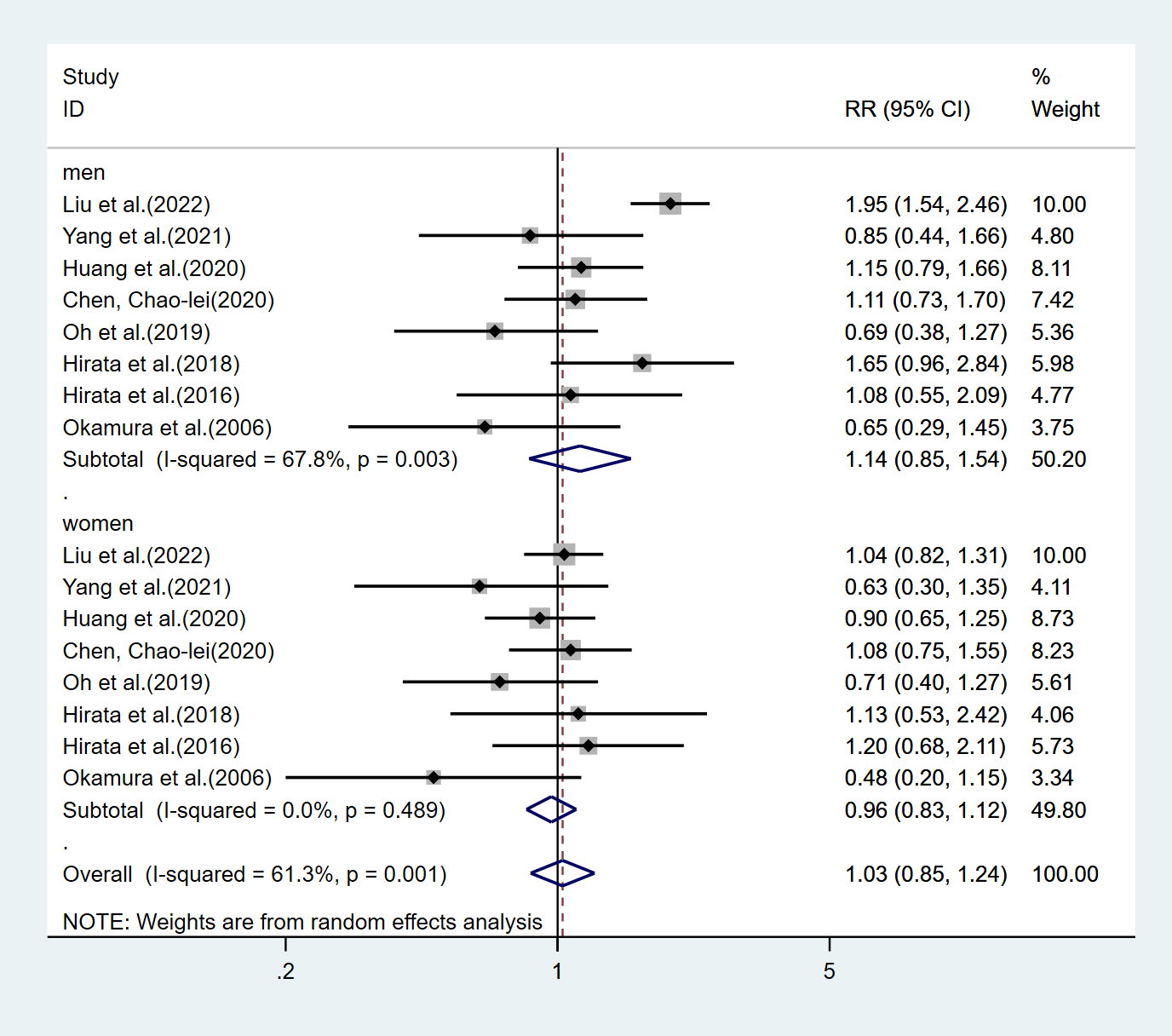


**Supplementary Figure S4.** Subgroup analysis of CVD mortality risk in men and women with very high HDL-C levels.


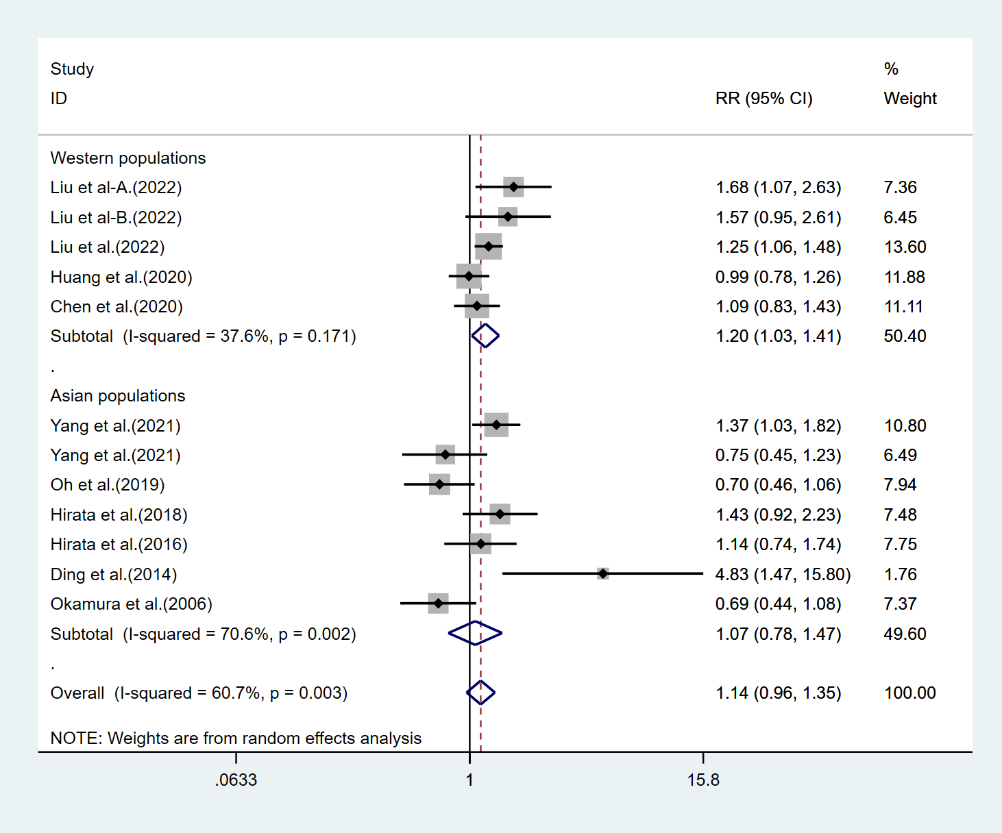


**Supplementary Figure S5.** Subgroup analysis of CVD mortality risk in Asian and Western populations with very high HDL-C levels.


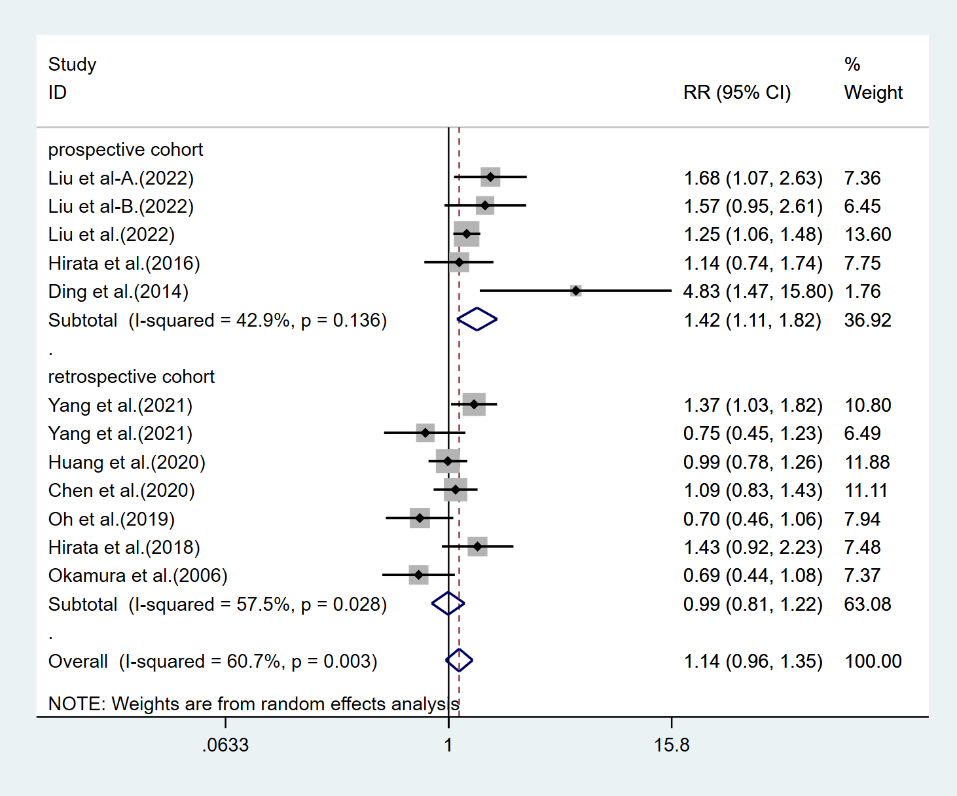


**Supplementary Figure S6.** Prospective and retrospective cohorts subgroup analysis of CVD mortality risk in patients with very high HDL-C levels.


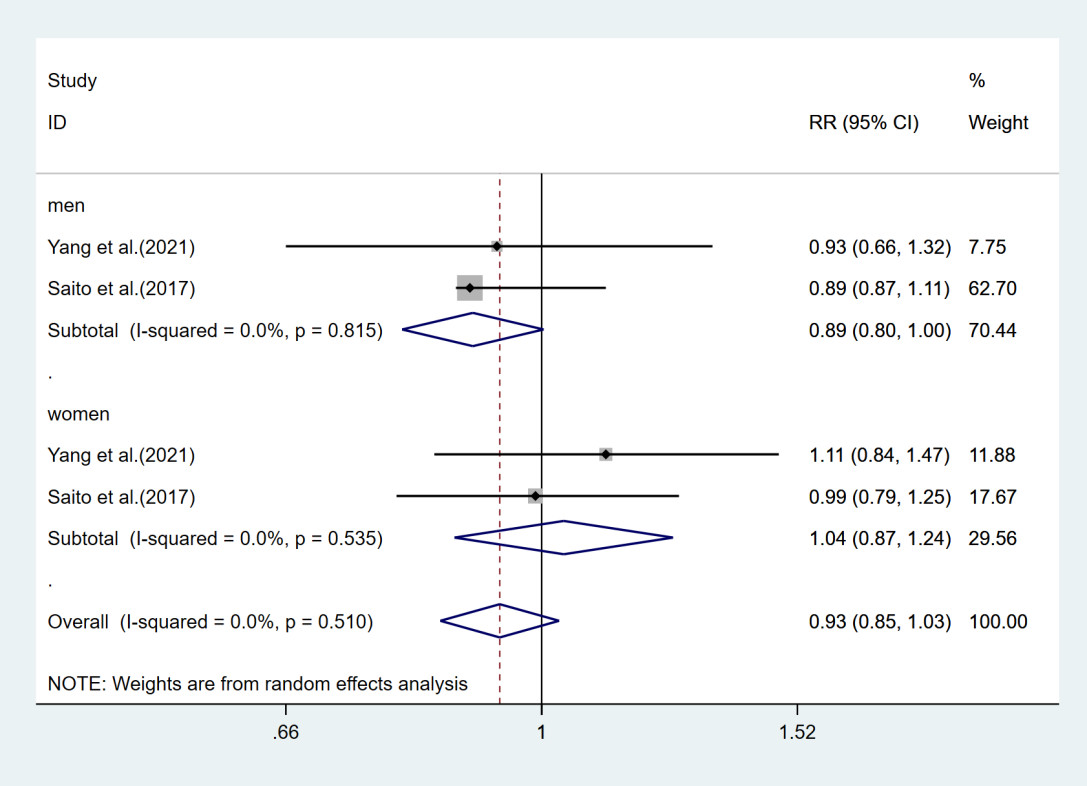


**Supplementary Figure S7**. Subgroup analysis of stroke risk in men and women with very high HDL-C levels.


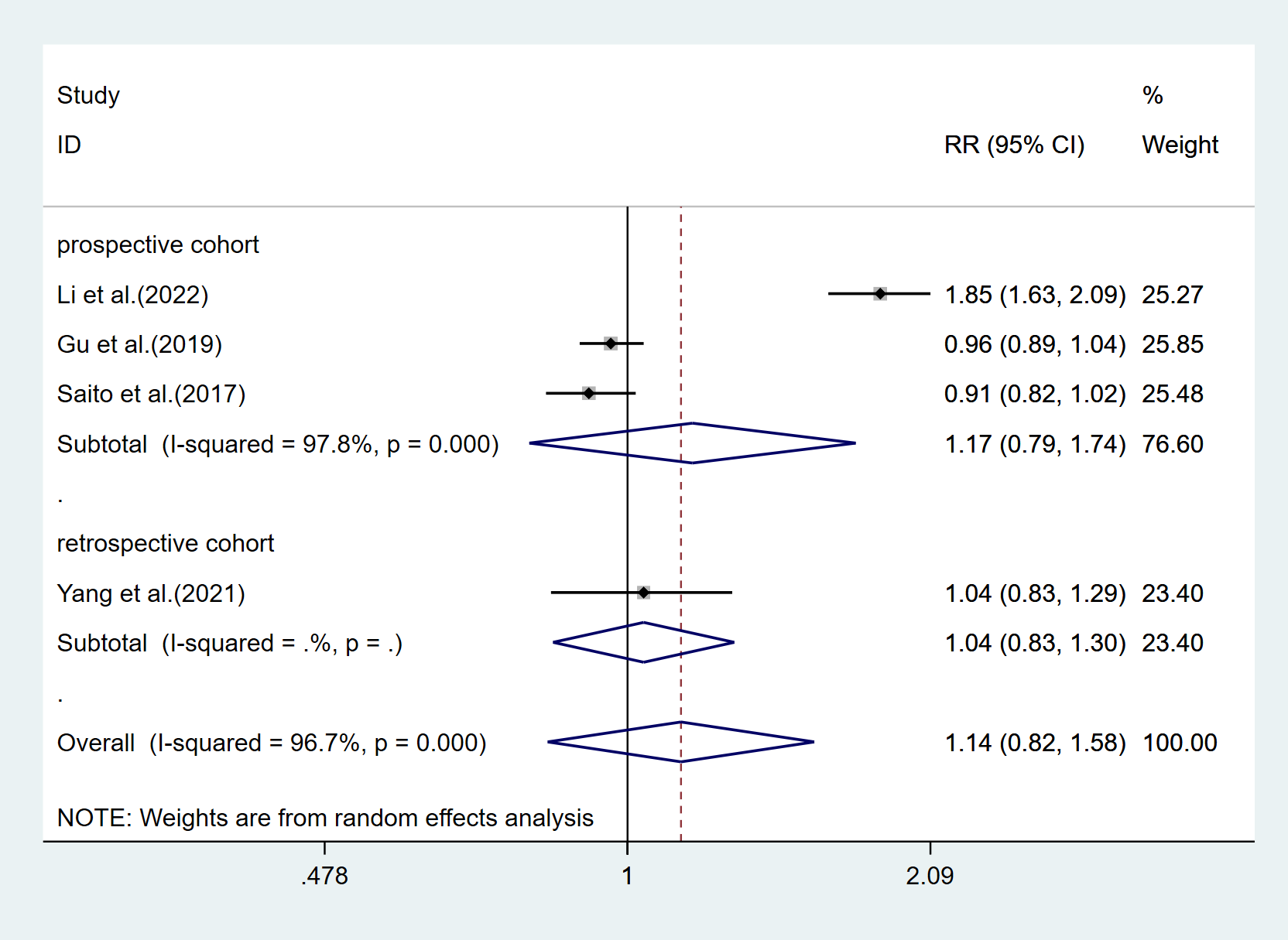


**Supplementary Figure S8.** Prospective and retrospective cohorts subgroup analysis of stroke risk in patients with very high HDL-C levels.

**Supplementary Figure S9.** Sensitivity analysis of all-cause mortality.

**Supplementary Figure S10.** Sensitivity analysis of CVD mortality.


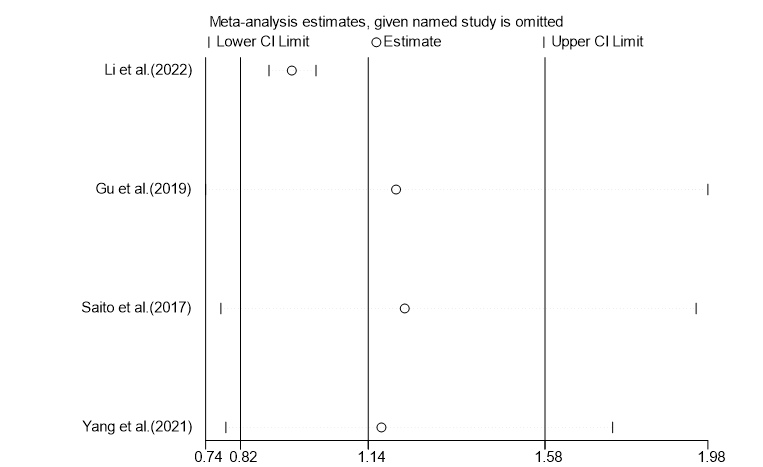


**Supplementary Figure S11**. Sensitivity analysis of Stroke.

## Supplementary Tables

**Supplementary Table S1.** Systematic literature review search terms and strategy.

| **Search terms for PubMed** |
| --- |
| #1 ("Cholesterol,HDL"[Mesh])OR "cholesterol,HDL"[Title/Abstract])OR"HDL" [Title/Abstract]OR "High Density Lipoprotein Cholesterol" [Title/Abstract]) |
| #2(“Mortality”[Title/Abstract]OR“Death[Title/Abstract]OR“Mortalities”[Title/Abstract]OR“Deaths”[Title/Abstract]OR”Fatal*”[Title/Abstract]OR“Cardiovascular”[Title/Abstract]OR“MACE”OR“Major Adverse Cardiovascular Events”OR“MACCE”OR“Major Adverse Cardiac and Cerebrovascular event”OR“Myocardial Infarction”OR“Myocardial Infarctions”OR“Cardiovascular Strokes”OR“Myocardial Infarcts”OR“Heart Attacks”OR“heart infarction)OR“Strokes”[Title/Abstract]OR“Cerebrovascular Accident”[Title/Abstract] OR“Cerebrovascular Accidents”[Title/Abstract]OR“CVA”[Title/Abstract]OR“CVAs”[Title/Abstract] OR“Brain Vascular Accidents”[Title/Abstract]OR“Apoplexy”[Title/Abstract]OR“Stroke”[Title/Abstract] OR“Heart Failure”[Title/Abstract]OR“Cardiac Failure”[Title/Abstract] OR“Heart Decompensation”[Title/Abstract]OR“Myocardial Failure”[Title/Abstract]) |
| #3 ("cohort Studies"[Mesh] OR “cohort analysis” [Title/Abstract] OR “follow-up” [Title/Abstract] OR “longitudinal” [Title/Abstract] OR “cohort ”[Title/Abstract] OR “prospective” [Title/Abstract] OR “retrospective” [Title/Abstract]) |
| #1 AND #2 AND #3 |
| **Search terms for Embase** |
| #1 high density lipoprotein cholesterol'/exp OR 'high density lipoprotein cholesterol':ab,ti OR hdl:ab,ti OR 'cholesterol, hdl':ab,ti |
| #2 mace:ab,ti OR 'major adverse cardiovascular events':ab,ti OR macce:ab,ti OR ('major adverse cardiac':ab,ti OR 'cerebrovascular event':ab,ti OR 'myocardial infarction':ab,ti OR 'myocardial infarctions':ab,ti OR 'cardiovascular strokes':ab,ti OR 'myocardial infarcts':ab,ti OR 'heart attacks':ab,ti OR 'heart infarction':ab,ti OR stroke:ab,ti OR strokes:ab,ti OR 'cerebrovascular accident':ab,ti OR 'cerebrovascular accidents':ab,ti OR cva:ab,ti OR cvas:ab,ti OR 'brain vascular accidents':ab,ti OR apoplexy:ab,ti OR 'heart failure':ab,ti OR 'cardiac failure':ab,ti OR 'heart decompensation':ab,ti OR 'myocardial failure':ab,ti cardiovascular:ab,ti OR mortality:ab,ti OR death:ab,ti OR mortalities:ab,ti OR deaths:ab,ti OR fatal*:ab,ti |
| #3 cohort studies':ab,ti OR 'follow up':ab,ti OR longitudinal:ab,ti OR cohort:ab,ti OR retrospective:ab,ti OR prospective:ab,ti OR 'cohort analysis':ab,ti |
| #1 AND #2 AND #3 |
| **Search terms for web of science** |
| #1 High Density Lipoprotein Cholesterol (Topic) or cholesterol, HDL (Topic) or HDL (Topic) |
| #2 Cardiovascular (Topic) or Mortality (Topic) or Death (Topic) or Mortalities (Topic) or MACE (Topic) or Major Adverse Cardiovascular Events (Topic) or MACCE (Topic) or Major Adverse Cardiac and Cerebrovascular event (Topic) or Myocardial Infarction (Topic) or Myocardial Infarctions (Topic) or Cardiovascular Strokes (Topic) or Myocardial Infarcts (Topic) or Heart Attacks (Topic) or heart infarction (Topic) or Stroke (Topic) or Strokes (Topic) or Cerebrovascular Accident (Topic) or Cerebrovascular Accidents (Topic) or CVA (Topic) or CVAs (Topic) or Brain Vascular Accidents (Topic) or Apoplexy (Topic) or Heart Failure (Topic) or Cardiac Failure (Topic) or Heart Decompensation (Topic) or Myocardial Failure (Topic) or Deaths (Topic) or Fatal* (Topic) |
| #3 High Density Lipoprotein Cholesterol (Topic) or cholesterol, HDL (Topic) or HDL (Topic) |
| #1 AND #2 AND #3 |
| **Search terms for Cochrane** |
| #1 MeSH descriptor: [Cholesterol, HDL] explode all trees |
| #2 (HDL):ti,ab,kw OR (High Density Lipoprotein Cholesterol):ti,ab,kw OR (cholesterol, HDL):ti,ab,kw |
| #3 (Cardiovascular):ti,ab,kw OR (Mortality):ti,ab,kw OR (Death):ti,ab,kw OR (Mortalities):ti,ab,kw OR (Deaths):ti,ab,kw OR (Fatal*):ti,ab,kw OR (MACE):ti,ab,kw OR (Major Adverse Cardiovascular Events):ti,ab,kw OR (MACCE):ti,ab,kw OR (Major Adverse Cardiac and Cerebrovascular OR (Myocardial Infarction):ti,ab,kw OR (Myocardial Infarctions):ti,ab,kw OR (Cardiovascular Strokes):ti,ab,kw OR (Myocardial Infarcts):ti,ab,kw OR (Heart Attacks):ti,ab,kw OR (heart infarction):ti,ab,kw OR(Stroke):ti,ab,kw OR (Strokes):ti,ab,kw OR (Cerebrovascular Accident):ti,ab,kw OR (Cerebrovascular Accidents):ti,ab,kw OR (CVA):ti,ab,kw OR (CVAs):ti,ab,kw OR (Brain Vascular Accidents):ti,ab,kw OR (Apoplexy):ti,ab,kw OR (Heart Failure):ti,ab,kw OR (Cardiac Failure):ti,ab,kw OR (Heart Decompensation):ti,ab,kw OR (Myocardial Failure):ti,ab,kw |
| #4 MeSH descriptor: [Cohort Studies] explode all trees OR (cohort studies):ti,ab,kw OR (follow up):ti,ab,kw OR (longitudinal):ti,ab,kw OR (cohort):ti,ab,kw OR (prospective):ti,ab,kw (retrospective):ti,ab,kw OR (cohort analysis):ti,ab,kw |
| (#1 OR #2) AND #3 AND #4 |

**Supplementary Table S2.** Characteristics of studies included in the meta-analysis.

| Author (year) | Country | Age (years) | Follow-up (years) | Sample size | Primary outcomes | Effect estimate | design | Subgroup | Comparison categories (mmol/L) and corresponding effect estimate (95% confidence interval) | Covariates in fully adjusted model | NOS |
| --- | --- | --- | --- | --- | --- | --- | --- | --- | --- | --- | --- |
| Liu,C.  (2022) | UK | 40-72 | 8.9 | 14,478 | All-cause mortality | HR | prospective cohort |  | 1.96(1.42-2.72) | age, sex, race and ethnicity, body mass,index,hypertension,diabetes, smoking, triglycerides, LDL-C, stroke history,heart attack history, eGFR, frequent alcohol use | 9 |
|  |  |  |  |  | Cardiovascular mortality |  |  |  | 1.68(1.07-2.63) |  |  |
| Liu,C.  (2022) | United States | ≥18 | 6.7 | 5,467 | All-cause mortality | HR | prospective cohort |  | 1.63(1.09-2.43) | age, sex, race,ethnicity, body mass index, hypertension,diabetes,current/former sm-oking,triglycerides,LDL-C,heart failure history,myocardial infarction history-y,eGFR,frequent alcohol use, statin use, aspirin use, β-blocker use,and angiote-nsinconverting enzyme inhibitor/angio-tensin receptorblocker use, HDL-C GRS plus top 10 principal comp-onents | 9 |
|  |  |  |  |  | Cardiovascular mortality |  |  |  | 1.57(0.95-2.61) |  |  |
| Liu,C.  (2022) | UK | 37-73 | 19 | 415,416 187,465 227,951 | All-cause mortality | HR | prospective cohort | all  men  women | 1.12 (1.03-1.20)  1.80 (1.59-2.03)  0.96 (0.87-1.05) | age, gender, race, body mass inde-x,hypertension,smoking,triglycerides,LDL-C,stroke history,heart attack history, diabetes,eGFR, frequent alcohol use, HDL-C GRS,and top 10 principal compo-nents | 8 |
|  |  |  |  | 415,416 187,465 227,951 | Cardiovascular mortality |  |  | all  men  women | 1.25 (1.06-1.48)  1.95 (1.54-2.46)  1.04 (0.82-1.31) |  |  |
| \| Li, H.  (2022) \| China \| 18-98 \| prospective \| 96 258 \| all \| 10 \| stroke \| HR \| 1.85(1.63-2.09) \| \| --- \| --- \| --- \| --- \| --- \| --- \| --- \| --- \| --- \| --- \| | China | 18-98 | 10 | 96,258 | stroke | HR | prospective cohort |  | 1.85(1.63-2.09) | age,sex,marital status,education, current smoking, drinking,average income,and regu-lar exercise, antihypertensive, hypoglycemic,and lipid-lowering medications,and updated cumulatively  averaged body mass index, systolic blood pressure,fasting blood glucose, hs-CRP,and estima-ted glomerular filtration rate | 9 |
| Yi, S. W.  (2021) | Korean | 19-99 | 8.6 | 69,163 | All-cause mortality | HR | prospective cohort | all men women | 1.28 (1.22-1.35)  1.30 (1.23-1.38)  1.21 (1.11-1.31) | age, sex , smoking status,alcohol use, physical activity, household incom-e,body mass inde-x,systolic blood p-ressure, fasting gl-ucose,triglyceride, known lipid disor-der,low density li-poprotein choles-terol | 9 |
| Yang,Zong-Ming  (2021) | China | ≥40 | 8.71 | 176,243 167,444 | All-cause mortality | HR | retrospective cohort study |  | 0.98 (0.78–1.22) 1.23 (0.96–1.58) | sex, marital status, educational level, tobacco smoking, alcohol drinking, physical activity, BMI, systolic pressure, LDL-C, heart diseases, cerebrovascular diseases, cancer, lipid-lowering therapy, anti-hypertensive therapy, glucoselow-ering therapy | 9 |
| Yang, Yeoree(2021) | Korean | ≥20 | 6 | 176,243 167,444 | All-cause mortality |  | retrospective cohort study | men women | 0.98 (0.78–1.22) 1.23 (0.96–1.58) | age, sex, body mass index, alcohol consumption, smoking, regular exercise, and income status, diabetes mellitus, hypertension, triglycerides, and use of lipid-lowering agents,HDL-C, high-density lipoprotein cholesterol | 9 |
|  |  |  |  |  | Cardiovascular mortality |  |  | men women | 0.85 (0.44–1.66)  0.63 (0.30–1.35) |  |  |
|  |  |  |  |  | stroke |  |  | men women | 0.93 (0.66–1.32) 1.11 (0.84–1.47) |  |  |
|  |  |  |  |  | MI |  |  | men women | 0.75 (0.47–1.21) 0.98 (0.65–1.48) |  |  |
| Huang, Yu-qing(2020) | United States | ≥18 | 15 | 42,145 20,415 21,630 | Cardiovascular mortality | HR | retrospective cohort study | all men women | 0.99（0.78,1.26) 1.15 (0.79–0.66) 0.90 (0.65–1.25) | age, gender, race, education level, smoking, body mass index, systolic blood pressure, estimated glomerular filtration rate, energy intake, total cholesterol, comorbidities (hypertension, diabetes, cardiovascular disease, and cancer), and medicine using | 9 |
|  |  |  |  |  | All-cause mortality |  |  | all men women | 1.14(1.02,1.22) 1.43 (1.21–1.70) 1.02 (0.88–1.17) |  |  |
| Chen, Chao-lei(2020) | United States | ≥18 | 15 | 11,497 | All-cause mortality | HR | retrospective cohort study | all men women | 1.20 (1.06, 1.37)  1.47 (1.20, 1.81)  1.09 (0.92, 1.28) | age, gender, race, education level, sm-oking, body mass index, energy, systolic blood pressure, estimated glomerular filtration rate, C-reactiveprotein, total cholesterol, diabetes, cardiovascular dis-ease, antihypertensive drugs, hypogly-cemic agents, antiplatelet drugs, lipidlo-wering drugs | 9 |
|  |  |  |  |  | Cardiovascular mortality |  |  | all men women | 1.09 (0.83, 1.43)  1.11 (0.73, 1.70)  1.08 (0.75, 1.55) |  |  |
| Oh, I. H.(2019) | Korean | >40 | 6 | 172,347 193,110 | All-cause mortality | HR | retrospective cohort study | men women | 1.17 (0.99–1.39) 1.12 (0.91–1.38) | age, systolic blood pressure, body mass index, fasting blood glucose, triglyceride, LDL cholesterol frequency of alcohol intake, smoking history,physical activity | 8 |
|  |  |  |  |  | Cardiovascular mortality |  |  | men women | 0.69 (0.38–1.27)  0.71 (0.40–1.27) |  |  |
| Li,X.  (2019） | China | ≥65,<98 | 8.76 | 100,070  79,913  20,157 | All-cause mortality | HR | prospective cohort | all men women | Ref 1.00; 1.05(0.93–1.18)  Ref1.00; 1.03(0.92–1.17)  Ref1.00; 1.21(0.80–1.81) | age, sex, education, physical activity, smoking, drinking, BMI, hs-CRP , non-HDL-C, triglycerides, chronic kidney disease, diabetes, hypertension, history of MI, history of stroke, history of mali-gnancy | 9 |
| Gu,Xiaoying  (2019) | China | ≥20 | 22 | 267,500 | Ischemic Stroke | HR | prospective cohort |  | 0.97 (0.89–1.06) | sex, age, smoking status, hypertension, geographic region, alcohol consumption, education level, and body mass index | 9 |
|  |  |  |  |  | Hemorrhagic Stroke |  |  |  | 0.94 (0.80–1.09) |  |  |
| Hirata, A.  (2018） | Japan | 40-90 | 12 | 43,407 21,108 22,299 | All-cause mortality | HR | retrospective cohort study | all men women | 1.15 (0.90–1.46)  1.24 (0.92–1.66)  1.01 (0.67–1.54) | age, body mass index, non HDL cholesterol, diabetes, hypertension, smo-king status, alcohol drinking status, and cohort. Sex was also adjusted in the men and women combined model | 9 |
|  |  |  |  |  | Cardiovascular mortality |  |  | all men women | 1.43 (0.92–2.23)  1.65 (0.96–2.84)  1.13 (0.53–2.42) |  |  |
| Saito, I.  (2017） | Japan | 40-69 | 15 | 10,571 20,165 | stroke | HR | prospective cohort | men | ref:1.00;1.12 (0.90-1.41) | age , smoking status, alcohol intake sports during leisure time, body mass index, menopause, systolic blood pressure, use of antihypertensive agents , diabetes, use of antilipemic agents, non-HDL chole-sterol | 8 |
|  |  |  |  |  |  |  |  | women | ref:1.00;1.01 (0.80-1.27) |  |  |
| Hirata, Aya  (2016) | Japan | ≥30 | 20 | 7,019 2,946 4,073 | All-cause mortality | HR | prospective cohort | all men women | 1.01(0.80,1.27) 1.02(0.73,1.43) 0.96(0.69,1.32) | age,BMI,hypertension,diabetes,smoking category,drinking category,non-HDLcholesterol,TG | 9 |
|  |  |  |  | 7,019 2,946 4,073 | Cardiovascular mortality |  |  | all men women | 1.14(0.74,1.74)  1.08(0.55,2.09)  1.20(0.68,2.11) |  |  |
| Ding, D.  (2014) | China | 40-85 | 5 | 1,916 | All-cause mortality | HR | retrospective cohort study |  | 3.06 (1.08–8.70) | age, gender, education, marriage, leisure-time physical activity, smoking, alcohol drinking, severity, duration, and treatment of CAD, history of diabetes, history of heart failure, BMI, systolic blood press-ure, glomerular filtration rate, and use of antihypertensive drugs, antidiabetic dru-gs, and antiplatelet drugs, use of chole-sterol-lowering drugs | 9 |
|  |  |  |  |  | Cardiovascular mortality |  |  |  | 4.83 (1.47–15.8) |  |  |
| Okamura, T.  (2006) | Japan | ≥30 | 9.6 | 8,384  3,504 4,880 | All-cause mortality | HR | retrospective cohort study | all men women | 0.70 (0.53, 0.93)  0.73 (0.50, 1.06)  0.63 (0.41, 0.94) | age, body mass index, trigfyceride ,non-HDL cholesterol, hypertension, diabetes, cigarette smoking category and alcohol intake category | 9 |
|  |  |  |  | 8,384  3,504 4,880 | Cardiovascular mortality |  |  | all men women | 0.56 (0.31, 1.01) 0.65 (0.29, 1.45)  0.48 (0.20, 1.15) |  |  |

CVD, cardiovascular disease; HR, hazard ratio; NOS, Newcastle-Ottawa Scale; Ref, reference; HDL High Density Lipoprotein Cholesterol

**Supplementary Table S3.** Newcastle-Ottawa Scale table.

| **study** | Selection | | | | Comparability | Outcome |  |  |  |
| --- | --- | --- | --- | --- | --- | --- | --- | --- | --- |
|  | Representativeness of the exposed cohort | Representativeness of the non-exposed cohort | Ascertainment of exposure | Outcome of interest not present at the start of the study | Comparability of the the exposed and non-exposed cohorts | Assessment of outcome | Sufficient follow-up time | Adequacy of follow-up | Total score |
| Liu,C.(2022)-A | 1 | 1 | 1 | 1 | 2 | 1 | 1 | 1 | 9 |
| Liu,C.(2022)-B | 1 | 1 | 1 | 1 | 2 | 1 | 1 | 1 | 9 |
| Liu, C.（2022） | 1 | 1 | 1 | 1 | 2 | 1 | 1 | 0 | 8 |
| LI,H(2022) | 1 | 1 | 1 | 1 | 2 | 1 | 1 | 1 | 9 |
| Yi, S. W.(2021) | 1 | 1 | 1 | 1 | 2 | 1 | 1 | 1 | 9 |
| Yang,Zong-Ming(2021) | 1 | 1 | 1 | 1 | 2 | 1 | 1 | 1 | 9 |
| Yang, Yeoree(2021) | 1 | 1 | 1 | 1 | 2 | 1 | 1 | 1 | 9 |
| Huang, Yu-qing(2020) | 1 | 1 | 1 | 1 | 2 | 1 | 1 | 0 | 8 |
| Chen, Chao-lei(2020) | 1 | 1 | 1 | 1 | 2 | 1 | 1 | 1 | 9 |
| Oh, I. H.(2019) | 1 | 1 | 1 | 1 | 2 | 1 | 1 | 0 | 8 |
| Li,X.(2019） | 1 | 1 | 1 | 1 | 2 | 1 | 1 | 1 | 9 |
| Gu,Xiaoying(2019) | 1 | 1 | 1 | 1 | 2 | 1 | 1 | 1 | 9 |
| Hirata, A.(2018） | 1 | 1 | 1 | 1 | 2 | 1 | 1 | 1 | 9 |
| Saito, I.(2017） | 1 | 1 | 1 | 1 | 2 | 1 | 1 | 0 | 8 |
| Hirata, Aya(2016) | 1 | 1 | 1 | 1 | 2 | 1 | 1 | 1 | 9 |
| Ding, D.(2014) | 1 | 1 | 1 | 1 | 2 | 1 | 1 | 1 | 9 |
| Okamura, T.(2006) | 1 | 1 | 1 | 1 | 2 | 1 | 1 | 1 | 9 |

Both A and B are the cohorts in the study of Liu, C. (2022). The two involved different regions and populations, and their study results are independent.
